# Investigating the outcomes of cardiopulmonary resuscitation and factors affecting it: A cross‐sectional study at Dr. Moaven Hospital, Sahneh City from 2014 to 2021

**DOI:** 10.1002/hsr2.1493

**Published:** 2023-08-17

**Authors:** Arash Ziapour, Vahid Hatami Garosi, Yasaman Tamri, Shohreh Ghazvineh, Ali Azizi

**Affiliations:** ^1^ Cardiovascular Research Center, Health Institute, Imam‐Ali Hospital Kermanshah University of Medical Sciences Kermanshah Iran; ^2^ Student Research Committee Kermanshah University of Medical Sciences Kermanshah Iran; ^3^ Kermanshah University of Medical Sciences Kermanshah Iran; ^4^ Department of Community Medicine, Faculty of Medicine Kermanshah University of Medical Sciences Kermanshah Iran

**Keywords:** cardiopulmonary arrest, cardiopulmonary resuscitation, success rate, underlying factors

## Abstract

**Background and Aims:**

Cardiopulmonary resuscitation (CPR) is referred to an attempt to maintain the respiratory system and blood circulation active to oxygenate the body's important organs until the heart and blood circulation system return to normal. CPR results are influenced by a variety of circumstances and factors. The purpose of this study was to look into the outcomes of CPR and the factors that influence them at the Dr. Moaven Hospital in Sahneh.

**Methods:**

This cross‐sectional descriptive study was carried out retrospectively from the start of 2014 to the start of 2021. Kermanshah University of Medical Sciences provides hospitals with a two‐page form for data collection. After entering the data into SPSS24, descriptive and inferential statistical tests were applied to analyze the results.

**Results:**

Out of 497 patients who referred to Dr. Moaven Hospital in Sahne City, 280 were men and 217 were women, with a resuscitation success rate of 22.5% in men and 23.5% in women. CPR was conducted on 63.2% of patients in the emergency department, with 22.2% of them having successful CPR. The existence of the underlying disease had a statistically significant link with the outcomes of CPR (*p* = 0.007). The most prevalent cause for visit was cardiorespiratory arrest (30.6%), and there was no statistically significant difference between the diagnostic and reason for visit and the outcome of resuscitation, according to the *χ* ^2^ test.

**Conclusion:**

According to the findings of this study, increasing age and duration of CPR, the existence of underlying diseases, and the absence of shockable rhythms all reduce the likelihood of success in CPR.

## 1 INTRODUCTION

Cardiopulmonary arrest, which can occur at any time and in any place, is among the most prevalent causes of death worldwide.^1^ Every year, Approximately 300,000 people in the United States suffer from out‐of‐hospital cardiac arrest.^2^ Despite considerable breakthroughs in the prevention of cardiac arrest, it is still regarded as the most serious health problem and the leading cause of death in the majority of countries around the world.^3^ Correct and rapid cardiopulmonary resuscitation (CPR) can reverse a vast number of cases of cardiopulmonary arrest. CPR of high quality improves the patient's chances of survival.^4^ However, many healthcare personnel are unable to execute optimal and high‐quality CPR, and CPR outcomes continue to be low globally.^4^, ^5^ Correct and principled CPR serves as the foundation for subsequent actions.^6^ In fact, successful CPR is dependent on treatments performed outside the patient's body to establish blood flow for oxygenation of the body's vital organs.^7^

Although the desired outcome of successful CPR is the patient's full return to life,^8^ people's survival rates are much lower, particularly in out‐of‐hospital resuscitation.^9^, ^10^ Various factors, which vary by country, are effective in CPR.^11^ The duration of the cardiorespiratory arrest until the initiation of CPR, underlying diseases, the availability of professional and trained resuscitation personnel, and the requisite tools and equipment are all effective factors.^12^ The place and time of the cardiorespiratory arrest are additional critical factors.^13^ In some research, the underlying cause is regarded as an important factor.^1^, ^14^ Age, gender, purpose for visit, use of defibrillation, and other characteristics all have an impact.^15^, ^16^ Age and gender cannot be changed, but factors such as minimal waste of time at the start of resuscitation operations, timely use of defibrillators, employees' level of knowledge and experience, and pharmaceutical interventions are among the factors that can increase the probability of patient survival with appropriate intervention.^15^, ^17^, ^18^ Investigating the factors influencing the success or failure of CPR is medically, economically, and ethically vital to propose adequate and scientific solutions to lessen the obstacles to successful resuscitation. As there have been few studies in this field in Iran, studies conducted in different climatic and social conditions may result in different outcomes, and other studies usually do not cover a long period of time and have a small number of samples, we conducted a study to Investigating the outcomes of CPR and factors affecting it: A cross‐sectional study at Dr. Moaven Hospital, Sahneh City from 2014 to 2021. We decided to conduct this study to determine the current conditions and to help improve the conditions and increase the rate of successful resuscitation by identifying and reducing negative influencing factors and improving and enhancing the strengths to increase the likelihood of patients returning to life.

## 2 MATERIALS AND METHODS

In this descriptive cross‐sectional study, 497 cases of CPR from the beginning of 2014 to the beginning of 2021 were examined retrospectively. During the 7 years, all patients with cardiorespiratory arrest who visited Dr. Moaven's Hospital in Sahneh City were studied. Patients who were not allowed to finish resuscitation by attendants and patients who just encountered respiratory arrest were excluded from the study. The data collection instrument was a two‐page form provided to hospitals by Kermanshah University of Medical Sciences that was filled out at the patient's bedside by supervisors who had been trained in this field and included demographic information about the patient, underlying disease, diagnosis, CPR process, drugs and equipment used, and the outcome of resuscitation, and so forth. Furthermore, a copy was filed in the nursing office where the researcher went to the research environment after obtaining the code of ethics and permission to begin research (990621) from Kermanshah University of Medical Sciences and used the registration forms for the resuscitation operation report and to access more complete information, the patients' files were referred to and the necessary information was extracted and recorded. After entering the data into the SPSS24, descriptive and inferential statistical tests were used to analyze the results.

## 3 RESULTS

**Figure 1:**
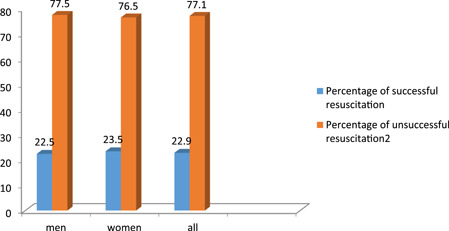
Rate of successful and unsuccessful resuscitation.

From the beginning of 2014 until the beginning of 2021, 497 patients visited the Dr. Moaven Hospital in Sahneh City, with 280 (56.3%) men and 217 (43.7%) women being evaluated. Men had a successful resuscitation rate of 22.5% and women had a rate of 23.5%. In all patients, 22.9% had successful resuscitation and 77.1% had unsuccessful resuscitation (Figure 1).

**Figure 2:**
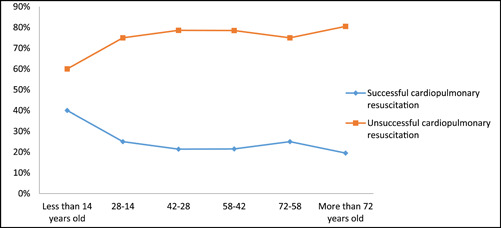
Outcomes of cardiopulmonary resuscitation based on age.

41.2% of patients were above the age of 72, with people aged 14−28 having the fewest visits (4.8%). There was a statistically significant relationship between age and resuscitation outcomes (*p* = 0.005) (Figure 2).

The impact of gender on resuscitation outcomes was not significant in this study. Eight percent of the patients who had successful CPR were resuscitated, with 27.5% having successful resuscitation. There was not a significant relationship between resuscitation history and the outcome of CPR. In the hospital, 68.2% of cases of cardiorespiratory arrest occurred, with 25% having successful resuscitation, and 31.8% occurring outside the hospital, with 18.3% having successful CPR. The *χ* ^2^ test revealed that there was no statistically significant difference between the location of the cardiorespiratory arrest (within or outside the hospital) and the outcome of resuscitation. CPR was conducted on 63.2% of patients in the emergency department, with 131 cases related to the general department of men. In maternity and pediatric departments, the least quantity of resuscitation was conducted (0.2%). The CPR success rate was 22.2% in the emergency department and 24% in other departments, with a statistically significant relationship between the unit that performed the resuscitation and the resuscitation outcomes (*p* = 0.007).

In terms of underlying diseases, 18.7% of the patients had high blood pressure, 16.9% had diabetes, 19.7% had cardiovascular diseases, 15.2% had other diseases, and 29.5% did not have any underlying disease, and there was a significant relationship between underlying disease and CPR outcomes (*p* = 0.007%). The patients' disease diagnosis and reason for the visit were divided into nine categories, which included cardiorespiratory arrest, cardiovascular diseases, neurological diseases, accidents and trauma, respiratory arrest, internal diseases, cancer, suicide, and the ninth category also included patients whose cause of cardiopulmonary arrest was in two or more groups. The most common reason for a visit was cardiorespiratory arrest (30.6%) and cardiovascular diseases (17.7%), while suicide (3.6%) was the least common reason for a visit. The most successful resuscitations were performed on suicide by poisoning (medicine and poison) (38/8), whereas the least successful were performed on accident and trauma patients (12/5). The *χ* ^2^ test revealed no statistically significant difference in the diagnosis, the reason for visit, and outcome of resuscitation. In 73/8 of the cases, the CPR operation began less than 1 min after the operation code was announced. 17.5% of CPR lasted less than 30 min, 69.6% lasted between 30 and 60 min, and 12.9% lasted longer than 60 min (Table 1).

**Table 1:** Resuscitation outcomes based on the duration of CPR operations.

Duration of resuscitation	Total	Successful resuscitation	Unuccessful resuscitation
Less than 30 min	17.5%	72.4%	27.6%
30−60 min	69.6%	12%	88%
More than 60 min	12.9%		
	Value	*df*	Asymp. sig (two‐sided)
Pearson's *χ* ^2^	146.16	2	0.000
Likelihood ratio	125.005	2	0.000
Linear by linear association	89.397	1	0.000
*n* of valid cases	497		

Abbreviation: CPR, cardiopulmonary resuscitation.

There was a statistically significant relationship between the duration of CPR and the outcome of CPR (*p* = 0.000). There was a statistically significant relationship between receiving a defibrillator and the outcome of resuscitation (*p* = 0.000) among the patients who received a defibrillator (55.4 had a final response to the defibrillator). At the time of admission, 77.7% of patients had no cardiac rhythm (asystole), 5.6% had PEA rhythm (pulseless electrical activity), 8% had VT rhythm, and 7.8% had VF rhythm. CPR was successful in 37.5% of patients with VT rhythm and 27.6% of patients with VF rhythm. The patient's cardiac rhythm at the time of admission had a statistically significant relationship with the outcome of CPR (*p* = 0.007).

## 4 DISCUSSION

Cardiac arrest is defined as a halt in blood circulation in which the heart muscle does not contract and there is no outflow or flow of blood. CPR is an attempt to artificially maintain the circulatory and respiratory systems working so that enough oxygen is delivered to keep the body's vital organs alive.^19^ The current study was designed to look into the outcomes of CPR and the factors that influence them at the Dr. Moaven Hospital in Sahneh City. According to the findings of this study, 280 of the patients were male, and 217 were female, with a rate of successful resuscitation of 22.5% in men and 23.5% in women. Examining the gender of the patients and its effect on the results of CPR was not significant, and Moeezi et al.^20^ found no significant difference in resuscitation success between men and women. According to Andersen et al., the success rate in resuscitation and hospital discharge was lower in women than in men.^21^ CPR was successful in 22.9% of patients and failed in 77.1%, according to one study, with an initial success rate of 58.91%.^22^ The initial success rate of CPR was reported as 36.7% in a study conducted by Nazri panjaki et al.^1^ According to some studies, the initial success rate of resuscitation is 25.4%.^15^ The reason for this center's lower success rate of CPR compared to other centers in the country is that some patients who came to the emergency room were completely dead, and due to cultural conditions and the insistence of the companions, the personnel were forced to perform resuscitation operations, which in some cases has resulted in a decrease in the overall success rate. About 41.2% of patients were over 72 years old, and the highest success rate was in the age group below 14 years and the lowest success rate was in the age group 72 years and older, demonstrating a relationship between age and resuscitation success rate. According to the findings of Lv JH and Hoybye's study, the success rate of resuscitation declined with increasing age, which can be linked to an increase in underlying diseases, natural physiological changes in the body, and old age.^23^, ^24^ Lima et al. discovered that younger patients have a higher chance of survival and spontaneous recovery of blood circulation after a shorter period of resuscitation.^25^ According to the findings of a study conducted by Moghadam Nia et al., there is not a statistically significant relationship between age and the success rate of CPR.^26^ There was not a significant relationship between the history of resuscitation and the results of CPR in 8% of the patients who were resuscitated more than once, which was in contrast to the study of Alizadeh et al. in which it was discovered that as the number of resuscitations for each patient increases, the final success rate reduces significantly, and this can be ascribed to complications produced by resuscitation.^15^ 68.2% of cases of cardiorespiratory arrest occurred inside the hospital and 31.8% outside the hospital, and according to the *χ* ^2^ test, there was no significant statistical difference between the place of the cardiorespiratory arrest (inside and outside the hospital) and the outcome of resuscitation. There was no significant difference in the success rate of resuscitation and cardiac arrest outside the hospital and cardiac arrest inside the hospital in the study of Moeezi et al.^20^ Miranzadeh et al. discovered that the survival rate in CPR in the hospital is lower than the survival rate outside the hospital.^27^ Patients who suffered cardiorespiratory arrest in the hospital had more successful resuscitation in the study of Darabian and Chen.^28^, ^29^ In this study, the general department of men had the highest success rate of CPR (27.4%), and there was a statistically significant relationship between the unit that performed resuscitation and the resuscitation outcomes. The success rate of resuscitation operations in emergency and specialty care departments differs statistically.^20^ According to Alizadeh et al., there was not a statistically significant relationship between the success rate of CPR and the portion of the body where the resuscitation was performed.^15^ The reason for more success in resuscitation operations in the general department of men compared to other departments of the hospital can be attributed to reasons such as the small number of patients in this unit and as a result more and more direct supervision and monitoring, as well as the better conditions of these patients compared to patients in the emergency department, special care, dialysis, and so forth. In line with the study of Alizadeh et al., in this study, cardiopulmonary arrest and cardiovascular diseases were the main causes of resuscitation.^15^ Among its causes, cardiovascular issues are intimately tied to the patient's life preservation. In contrast to this study, the majority of patients who underwent resuscitation in the studies of Montazer et al., Nazri panjaki et al., and Jafarian et al. were patients with internal diseases.^1^, ^30^, ^31^ Patients with poisoning had the highest rate of success in resuscitation, while trauma patients had the lowest rate of success. The success rate of CPR was higher in poisoning patients in the study of Jafarian et al.^31^ There was no statistically significant relationship discovered in the current study between the cause of cardiopulmonary arrest in patients and the outcomes of resuscitation. In line with this study, Nazri Panjeki et al.^1^ found no statistically significant link between the cause of cardiopulmonary arrest and the outcomes of resuscitation operations. In contrast to the findings of this investigation, there was a statistically significant relationship between primary causative diseases and the outcome of CPR in the study of Alizadeh et al.^15^ Regarding the effectiveness of underlying disease on the outcomes of CPR in this study, 29.5% of the patients or their companions did not state any underlying disease, and the success rate of CPR was significantly higher in these patients compared to patients with underlying disease. The existence of an underlying disease raises the failure rate of resuscitation and decreases the survival rate following resuscitation, as found in Chan and Esfahani's study.^14^, ^19^ Despite the existence of varied outcomes, the effect of underlying disease as an outcome of CPR was not statistically significant in the study of Nazri panjaki et al.^1^ Analyzing the duration of successful and unsuccessful resuscitation is critical, and most patients received CPR less than 1 min after the code was announced. There was a statistically significant relationship between the duration of resuscitation and the outcome of CPR in 72.4% of the patients whose resuscitation duration was less than 30 min. Various studies show that the duration of CPR is related to the return of spontaneous circulation, that the greatest benefits of CPR can be obtained in the first 10−15 min, and that increasing the duration of CPR will bring minimal benefits, with the average duration of CPR in patients who had successful CPR being less than 30 min.^32^ According to Albinali et al., the average duration of CPR in patients who survived was 26.5 min, which is similar to the current study.^33^ Receiving a defibrillator had a statistically significant relationship with the outcome of CPR. 37.5% of VT patients who received a defibrillator returned to their cardiac rhythm and had a successful resuscitation, while 27.6% of Vf patients who received a defibrillator had a successful resuscitation. According to Andersen and Kanori,^21^, ^34^ patients with shockable rhythms are two to three times more likely to return and live than patients without shockable rhythms.

One of the limitations of this study is the lack of investigating the effect of performing basic CPR by companions before transfer to the hospital. Because there was no statistically significant relationship between the place of cardiorespiratory arrest (inside or outside the hospital) and the outcomes of CPR, it is suggested that a study be conducted taking into account companions' ability to perform basic CPR and the effect of performing basic resuscitation before transferring the patient to the hospital on the success rate of resuscitation operations.

## 5 CONCLUSION

It is possible to conclude that factors such as the growing age and duration of CPR, the existence of underlying diseases, and the absence of shockable rhythms reduce the likelihood of success in CPR. Given the lower rate of success of CPR compared to other centers both within and outside the country, it is suggested that the reasons for resuscitation operation failure can be found in other factors such as the knowledge and ability of the resuscitation team personnel, the personnel's experience, and so on.

## AUTHOR CONTRIBUTIONS

**Arash Ziapour**: Conceptualization; investigation; writing—original draft; writing—review and editing. **Vahid Hatami Garosi**: Conceptualization; writing—original draft; writing—review and editing. **Yasaman Tamri**: Methodology; resources; software. **Shohreh Ghazvineh**: Data curation; validation. **Ali Azizi**: Project administration; supervision; visualization.

## CONFLICT OF INTEREST STATEMENT

The authors declare no conflict of interest.

## ETHICS STATEMENT

The undertaken procedures were approved by the Medical Ethics Committee of Kermanshah University of Medical Sciences IR.KUMS.REC.1398.320. Consent to submit has been received explicitly from all coauthors, as well as from the responsible authorities—tacitly or explicitly—at the institute/organization where the work has been carried out, before the work is submitted. The purpose of this research was completely explained to the participants through the cover page of the questionnaire, and they were assured that their information would be kept confidential by the researcher. Informed consent from the participants was acquired as they agreed to participate in the study by reviewing the questionnaire's cover page and clicking on the provided link. Furthermore, for participants younger than 18 years of age, the participant was asked for the consent of the parent or guardian.

## TRANSPARENCY STATEMENT

The lead author Arash Ziapour affirms that this manuscript is an honest, accurate, and transparent account of the study being reported; that no important aspects of the study have been omitted; and that any discrepancies from the study as planned (and, if relevant, registered) have been explained.

## Data Availability

All the data are available from the authors through reasonable request.
